# Obscured-ensemble models for genomic prediction

**DOI:** 10.1371/journal.pone.0334239

**Published:** 2025-11-14

**Authors:** Rounak Saha, Amir Morshedian, Jia Sun, Robert Duncan, Mike Domaratzki

**Affiliations:** 1 IIT Kharagpur; 2 Department of Computer Science, Western University; 3 Department of Plant Science, University of Manitoba; University of North Texas, UNITED STATES OF AMERICA

## Abstract

Genomic Prediction (GP) uses dense whole-genome marker sets from lines of a crop to predict agronomic traits for untested genotypes. In recent years, deep learning (DL) approaches for genomic prediction have demonstrated state-of-the-art results. However, substantial variation exists in DL outcomes for GP as the success of DL is dependent on the architecture of the model used, as well as the amount of data available and the population structure of the individuals in the training set. In this paper, we consider an obscured model for GP, where the model is not provided with genomic content. The obscured model was intended to evaluate the possibility of so-called shortcut learning in GP.We conclude that we can perform GP using the obscured model with only 20% of the obscured markers from each reference genotype. This selective feature usage significantly enhances the efficiency of our model without compromising accuracy. By eliminating markers, we demonstrate that the model is not relying on linkage to perform shortcut learning. Further, we consider a deep learning ensemble method for genomic prediction based on the obscured model. The ensemble model we develop here shows success as a method for GP by using the similarity to each of the elements of a training set of genotypes, as well as the performance of the genotypes. We evaluate the obscured ensemble model for GP. We demonstrate that the obscured ensemble model is successful even with a limited number of genotypes used for prediction. Further, random selection of a subset of genotypes is sufficient to ensure successful performance.

## Introduction

Genomic Prediction (GP) is an important tool in modern breeding programs. GP uses sets of dense whole-genome markers from several genotypes of a species to predict a phenotype [1]. With current data sets consisting of hundreds to thousands of genotypes and tens of thousands of genomic markers, efficient techniques that accurately predict breeding values are essential. The benefits of GP are well-documented [2]: employing GP can reduce breeding cycles and breeding costs. Additionally, GP allows targeting of complex polygenic traits. This allows the development of elite cultivars with improved agronomic performance, nutritional quality, or disease resistance.

Many different approaches have been used for GP, including linear models such as Best Linear Unbiased Predictor (BLUP) and related versions such as GBLUP, and Bayesian methods which extend linear models by imposing different priors on marker effects, offering robust performance in cases of smaller sample sizes or sparse marker effects. Additionally, machine learning models [3–5] have been studied, which includes many supervised approaches, including ensemble techniques like Random Forests (RFs) and Gradient Boosting Machines (XGBoost) [6,7]; kernel methods such as Support Vector Machines (SVMs) [8] and Recurring Hilbert Kernel Spaces (RKHS) [9]; and deep learning (DL) methods [4,5] have demonstrated strong predictive capabilities by capturing complex nonlinear interactions.

Overall, research has demonstrated that no one single prediction method dominates in GP, and that different models have different performance even on the same dataset [3–5]. Research has demonstrated that no single model consistently outperforms others, as performance variations exist even for traits within the same dataset. These differences can arise from factors such as population structure, trait heritability, and training set size [2,10]. Linear models such as BLUP and GBLUP demonstrate continued strength in modelling even in comparison to more complicated DL models [11]. Combined with the relative simplicity of using these models, they remain popular choices in GP. However, DL models continue to be examined due to their capacity to easily handle multiple heterogenous data sources and nonlinear interactions.

There has been significant recent research into the use of DL models for GP in single environment models [4,5,11–14]. DL models are of particular interest in GP as they are capable of modelling nonlinear effects in traits (e.g., epistasis or dominance). DL models typically consist of neural networks, where marker data is fed into a series of layers of nodes. The nodes compute simple functions to extract higher level patterns, which are then passed to subsequent layers. Ensemble models have also been employed in GP [6,7,15]. Ensemble methods use a collection of simple learners to make independent predictions and then combine these to achieve a final prediction.

To ensure a more comprehensive evaluation, we emphasize the importance of examining both Pearson correlation coefficient (PCC) and Mean Squared Error (MSE) when assessing predictive models for GP. While PCC quantifies the linear agreement between predicted and observed values, MSE captures the magnitude of prediction errors, offering deeper insight into model efficiency.

In a recent paper, Ubbens et al. [16] demonstrated that GP using DL may be subject to so-called shortcut learning [17]. DL models that predict phenotype may be learning attributes of the data unrelated to the values of the features (i.e., the genomic markers). The results of Ubbens et al. demonstrate that the actual content of the markers is not needed to make predictions, and instead the relative similarity of genotypes or position of the markers can be the source of the predictions. This method is referred to as an *obscured model*, as the actual content of the markers is obscured and replaced, as input to the DL model, but the similarity of a pair of genotypes.

The operation of the obscured model of Ubbens et al. is analogous to the operation of GBLUP, one of the most common approaches for GP, where the similarities for each pair of genomes are computed as numerical values and stored in the genomic relatedness matrix (GRM). In the Ubbens et al. model, the numerical value representing the similarity is replaced with a binary vector, described below. This vector is then used as a part of the input to a DL model.

Despite the growing interest in deep learning for genomic prediction, challenges such as interpretability and robustness remain. Shortcut learning raises concerns regarding the reliability of DL-based predictions, particularly in breeding programs where accurate selection decisions are essential. Understanding the extent to which genomic markers contribute to predictive accuracy versus dataset artifacts is crucial for ensuring the reliability of modern genomic prediction models.

In this paper, we consider two aspects of this obscured ensemble model of Ubbens et al. We first consider the relative performance of the obscured model as a predictor under the assumption of shortcut learning. We consider feature selection to reduce the amount of genomic data used in the prediction. We do this in the context of the potential role that linkage may have on shortcut learning, and whether the density of markers in modern GP data sets is a potential source of the shortcut learning noted by Ubbens et al. We hypothesize that linkage between markers may provide a source of shortcut learning: when markers are dense, linkage between genomic markers may allow information about important variation to persist in the data, even in the context of the obscured information.

In this work, we describe what we call an *obscured ensemble method*, which is an ensemble model that uses the obscured model of Ubbens et al. as the base model. The different obscured base models of the ensemble method differ in which pairs of genomes are compared to build a consensus value for the final phenotype prediction. We investigate the performance of the obscured technique not for evidence of shortcut learning, but as a predictor in its own right. We consider the performance of the obscured ensemble model as a predictor, and whether the efficiency can be improved by reducing the number of elements used to obtain the average. We hypothesize that a targeted set of instances for comparison, obtained by the best overall performance on the training set, can be chosen to drive the ensemble model. By systematically evaluating obscured ensemble methods, this study provides insights into the possible model efficiency.

## Background

### Neural networks for genomic prediction

Neural networks are a type of artificial intelligence inspired by how the human brain processes information. Just as our brain consists of interconnected neurons that work together to recognize patterns—such as identifying a face or understanding speech—a neural network learns patterns from data to make predictions. These models are particularly useful when dealing with complex relationships, like those found in genomic data, where multiple genetic markers interact in ways that influence traits.

**Fig 1:**
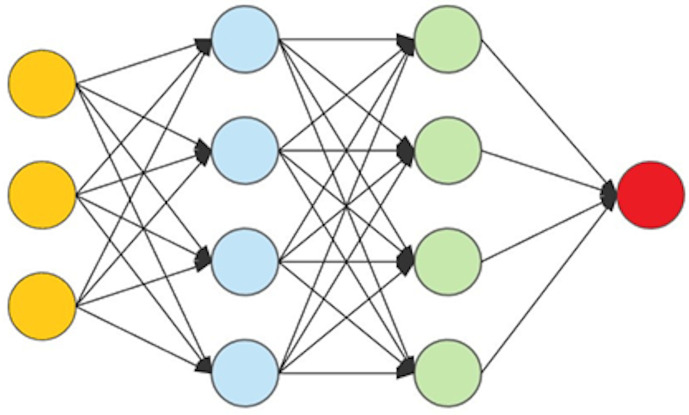
Structure of a neural network. A feedforward neural network with an input layer (yellow), hidden layers (blue and green), and an output layer (red). Arrows indicate the flow of information through weighted connections.

A neural network consists of a collection of nodes that accept numerical data from either other nodes or from the input data. The nodes then perform a relatively simple computation that calculates an output value, which is then transmitted to the next layer of the network. Nodes are organized into the input layer (which receives input), followed by a series of hidden layers, and then finally an output layer (see Fig 1).

The computation at each node is a weighted sum of the values of the nodes that feed into that node. Thus, if the values $x_{1},x_{2},\ldots ,x_{n}$ are output from the nodes in the previous layer, then the node computes the value $y=b+\sum_{i=1}^{n}w_{i}x_{i}$, where the values $w_{i}$ are the network weights and b is the bias term. These values are learned through the neural network training process, where a training set of data and outputs are provided to the network and the values of the weights are updated. Additionally, an activation function is applied to y  before broadcasting it to the next layer of nodes. The activation function, typically a function such as sigmoid, tanh or popularly the ReLU function, adds a capacity for non-linear calculations that are essential to the power of neural networks. For more details on these details, especially as they relate to the model employed in this paper, please see Ma et al. [11].

Neural networks are referred to as “deep” when they contain several hidden layers. Neural networks have been employed in genomic prediction for many crops [5,18]. Several variations of deep networks, which differ based on the configuration of the layers and types of nodes, have been used, including fully connected networks [7,19], convolutional neural networks [11], and, more recently, transformer models [12,20].

### Feature selection in genomic prediction

Feature selection, where the set of features of a data set is pruned to discard those that are not expected to affect prediction, is an important aspect of the design of machine learning problems. Feature selection is particularly relevant in GP [21,22]. In GP, the number of features is often in the tens of thousands, while the number of instances (i.e., the number of phenotyped lines) is typically in the hundreds to thousands. This situation makes GP especially prone to overfitting by machine learning models but feature selection can mitigate this overfitting.

Many feature selection approaches in GP in the literature have been from the broad category of filter methods [22,23]. A major motivation for the use of these methods is efficiency. Filter methods evaluate each feature independently to determine if a linear relationship exists with the trait. As GP is typically formulated as a regression problem, measures such as mutual information [11] and chi-squared test [23] have been used. Random selection of features has also been considered [10,24]. It has been shown that filter-based feature selection outperforms random selection for GP using DL models [23]. Research has also investigated the use of GWAS as a filter feature selection method in GP [25–27].

### Shortcut learning

In many real-world scenarios, deep learning models can rely on unintended correlations rather than learning meaningful patterns. For example, a model for recognizing animals in photos might associate cows with green fields and blue skies that may commonly appear in pictures of cows in the training set. Such a model may predict an image to show a cow by recognizing these environmental backgrounds rather than any specific attributes of the animal itself. This could happen if most images labeled “cow” share the same outdoor setting, leading the model to rely on grass and sky as identifying features instead of learning the actual shape or characteristics of the cow. This phenomenon is known as shortcut learning.

Similarly, in genomic prediction, deep learning models may not be learning the effects of specific genetic markers but rather exploiting overall genetic similarity. Ubbens et al. [16] demonstrate this by showing that a model can predict an individual’s trait using only the locations of matching markers between two genotypes, without access to actual marker values. This suggests the model is leveraging genetic relatedness as a shortcut rather than interpreting true biological signals.

**Fig 2:**
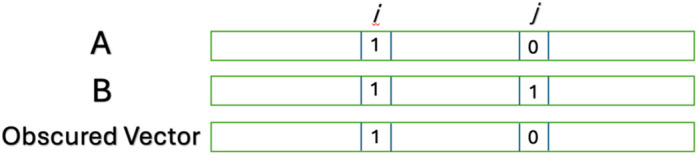
Construction of the obscured vector from two genotypes A and B.

Specifically, Ubbens *et al.* [16] demonstrated that GP using DL may be subject to shortcut learning [17]. Ubbens *et al.* demonstrate that, for two genotypes *A* and *B*, with observed values *y*_*A*_ and *y*_*B*_ for a single trait (e.g., yield measurements for genotypes A and B), when their model is provided with the trait y_B_ and only the points of similarity between *A* and *B*, a model can predict *y*_*A*_, with accuracy comparable to a standard DL-based GP approach. The results demonstrate that the actual content of the markers is not used to make prediction, and instead the relative similarity of genotypes or position of the markers may be the source of the predictions. The points of similarity between *A* and *B* are modeled by an obscured vector. Suppose that the number of markers in genotype A and B is *n*. Then the obscured vector also has a length of *n*, and the value of the obscured vector at position *i* is 1 if *A* and *B* agree on the marker at position *i*, and zero otherwise, as shown in Fig 2.

To predict the trait of *A* (i.e., the genetically estimated breeding value)*,* the model is provided with the obscured vector between *A* and another genotype *B,* as well as the phenotypic trait value for *B*. The novelty of the obscured model is that it makes this prediction without any reference to the content of the genotype of *A*: it learns the relationships without knowledge of the markers of the genotype of *A*, including the relationship between the markers (e.g., dominance).

Evaluating the success of this model is challenging as predicted traits for a genotype can only be obtained in relation to the other genotype (i.e., *B*). To evaluate the performance of their model on a genotype *g*, Ubbens *et al.* [16] use the mean prediction of each genotype in the training set when paired with *g*.

**Fig 3:**
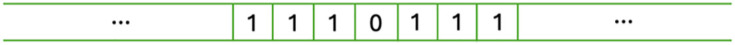
Neighbourhood of an informative SNP in an obscured vector v.

First, we consider the potential for linkage to be a source of the shortcut learning noted by Ubbens *et al.* Consider, for example, two genotype instances *A* and *B* and an informative SNP for a trait at a position *x*. If nearby SNPs in LD with the SNP at position *x* is also included in the model, then these may provide an avenue for shortcut learning. Consider the scenario where we are providing the model with the obscured vector v for *A* and *B*, and the phenotype for B. Suppose the immediate neighbourhood of position x in the vector v is as shown in Fig 3 below.

In this case, when provided with the obscured vector v, the model may learn that an important mutation exists between *A* and *B* at position x, while differences do not exist in adjacent positions. This may lead the model to learn that the phenotype for *A* should be substantially different from that of *B*. However, by reducing the density of markers, including by removing those markers that are in LD with others, we can determine if shortcut learning still exists.

Additionally, we note that the final prediction of the model developed by Ubbens *et al.* acts as a form of ensemble learning: the evaluation of *g* with every element of the training set is used, and the final value is taken as the mean of these predictions. This is an common approach for regression problems for ensemble models [28]. Thus, we investigate the performance of this technique not for evidence of shortcut learning, but as an ensemble method. We call this overall ensemble model an obscured ensemble method.

**Fig 4:**
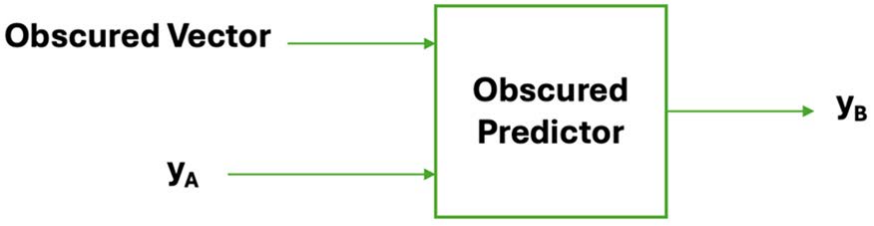
Overview of the obscured model proposed by Ubbens et al.

Fig 4 illustrates the concept of the obscured predictor model as proposed by Ubbens et al. In this framework, the model does not rely directly on genomic marker values but instead learns patterns based on the similarity structure of the data, potentially due to shortcut learning. The predictor, represented as an ‘obscure predictor,’ receives input from two genotypes, *A* and *B* and makes predictions based on obscured features rather than meaningful genetic variation. If the obscured representation consists entirely of 1s, for example, then the genotype of A and B are the same (at the sampled locations). In this case, the model would tend to assign the same prediction to both genotypes (*y*_*A*_ = *y*_*B*_). Conversely, variations in the genotype of A and B would result in an obscured representation with more zeros in the feature space, which can lead the model to distinguish between genotypes, even when actual genetic content is not explicitly considered. This would lead the model to predict a difference between y_A_ and y_B_. This highlights a key challenge in deep learning-based genomic prediction, where models may exploit unintended patterns in the data rather than true biological signals.

### Ensemble methods

Ensemble approaches for machine learning, such as random forests and gradient boosting, have shown strong results in machine learning. These have been employed in GP [6,7,15]. These models are characterized by collections of simpler machine learning models, such as decision trees, which we call the base model. The training methods for these models differs, but techniques are employed to ensure each base model does not produce the same result for an input. This can include allowing only partial information for an input instance for each element of the ensemble collection.

For a new input, each element of the collection produces an independent prediction. Then a final prediction for the input is obtained by consulting the predictions of the elements of the collection. In the case of regression models, which are common in GP, these individual predictions are typically averaged to produce the predicted value.

We describe below an obscured ensemble method for GP. This uses the Ubbens et al. obscured model as the base model for our ensemble method. By averaging the predictions of the obscured model over several different pairs of individuals, we produce an overall prediction for the obscured ensemble model.

## Materials and methods

### Data sets

Two data sets were examined. The first is a canola (*Brassica napus* L.) data set, described in [29]. The data set contains 459 genotypes with 16,166 features. The data set consists of a founder population and a set of F_1_ hybrids. The traits of interest are yield, protein content and oil content.

A publicly available lentil (*Lens culinaris Medik.*) dataset [30] has also been considered. This data set was also used by Ubbens *et al.* [16]. The diversity panel contains 324 genotypes with 13,304 markers. The trait of interest is Days to Flowering. This trait was considered by Ubbens et al., who demonstrated that their obscured model performed comparably to an established DL model, DeepGS, on this trait. For both data sets, trait values were standardized by using z-scores.

### Deep neural network structure

**Fig 5:**
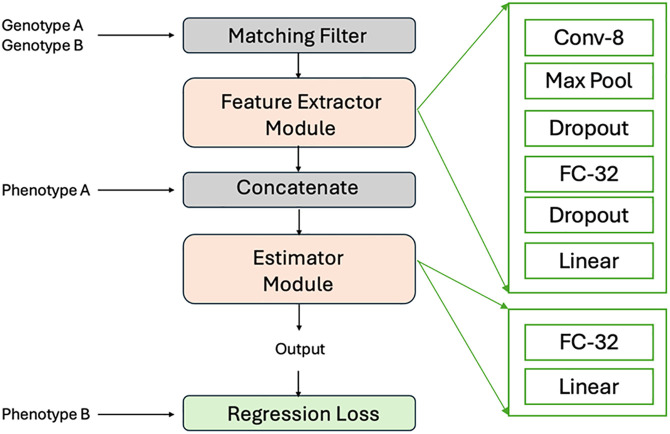
Overview of the obscured model for phenotype prediction without marker data access. Based on Ubben et al. [16] and incorporating feature extractor modules from Ma et al. [11], the pipeline aligns genotypes via a matching filter and processes them with a DeepGS-based network for feature extraction and estimation.

We now describe the neural network architecture used throughout this paper. The basis of the predictions using the obscured vectors is a deep learning module modified from an original design for standard GP. Fig 5 presents the architecture of the obscured model designed for training a deep neural network without direct access to marker data. This pipeline, based on Ubben et al. [16] and incorporating feature extractor modules based on Ma et al. [11]**,** integrates key components to predict phenotypes from genotypes:

1. **Matching Filter**: The initial stage of the model preprocesses and matches genotypic data from Genotype A and Genotype B before feeding it into the neural network, creating the obscured vector that serves as input for prediction.
2. **Feature Extractor Module**: This module employs the DeepGS architecture, originally proposed by Ma et al. [11]. It includes several layers designed to reduce the dimension of the features in the obscured vector for efficiency. The module creates a low-dimensional representation of the differences between genotypes A and B which were provided in the obscured vector as input to this module.
3. **Concatenate**: This step combines the features extracted from the genotypes with the phenotypic data (Phenotype A), preparing it for the estimation process.
4. **Estimator Module**: Consists of a two-layer fully connected network that utilizes the combined data to estimate the target output. This module refines the prediction through layers designed to interpret the integrated data. The output of this module is the predicted Phenotype B.
5. **Regression Loss**: The output from the Estimator Module is compared with the actual Phenotype B to compute the regression loss.

The overall training and prediction process of the Ubbens et al. model can be considered to have a training set that consists of (a) a matrix with rows that consist of the concatenation of an obscured vector (for a possible pair of genotypes) and one of the phenotypes, as well as (b) a vector of the remaining phenotype. The model will then learn the parameters to be able to predict the remaining phenotype from the (obscured vector, phenotype) data. The success of the model can be evaluated on a test set of the same matrix and vector format. In comparison to a traditional GP model, the Ubbens et al. model does not take a single individual to predict its phenotype. Rather, the model is responsible for learning how the differences in the obscured vector are reflected in differences in the phenotype (i.e., numerical differences between phenotype A and phenotype B).

However, the model of Ubbens et al. can also be considered from the perspective of, e.g., Genotype B and Phenotype B only, to obtain a prediction in a similar way to how a typical ML model would. We describe this process, as our obscured ensemble model, below.

### Examining effects of feature selection

To assess how the obscured model relies on similarity between adjacent features, we explored feature selection to reduce feature density. First, we applied a pairwise feature selection approach to remove redundant features. This was done using a sliding window method: for a fixed integer w, each feature was compared with the next w features, and any identical features within the window were eliminated. Experiments with different values of w showed that beyond w = 8, the number of identified redundant pairs remained stable.

To evaluate whether feature selection contributes to shortcut learning, we implemented multiple machine learning (ML) and classical models. The obscured model from Ubbens et al. was implemented using the authors’ provided code, which is based on the DeepGS model of Ma et al. [11]. For comparison, we also tested the original DeepGS model, along with Random Forest (RF), GBLUP and Ridge Regression. Like Ubbens et al., the obscured model was trained in an end-to-end manner for 20 epochs. In this way, the model learns the parameters of the layers to allow prediction of the phenotype y_B_ from the obscured vector and y_A_.

After removing redundant features, we applied mutual information-based feature selection to retain only the most relevant features for predicting the trait of interest. The number of retained features varied from a minimum of 8 up to the full set of remaining features after redundancy filtering. The performance of all models (Obscured, DeepGS, Ridge Regression, RF, and GBLUP) was compared across different feature set sizes.

To ensure a fair evaluation, we used three-fold cross-validation with consistent folds across all models.

### Obscured ensemble methods for phenotype prediction

**Fig 6:**
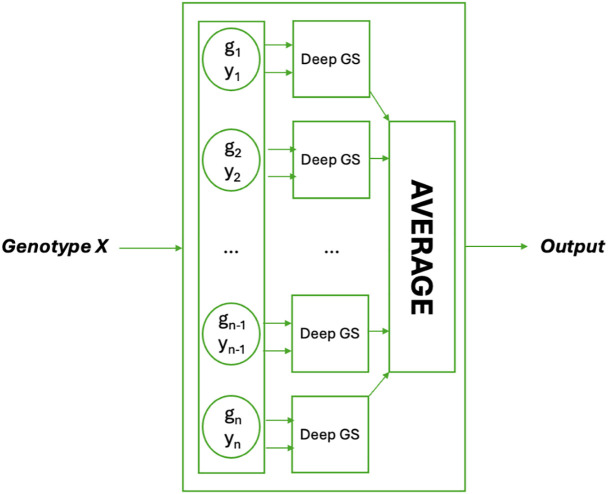
The Obscured Ensemble Model. A single genotype X is paired with multiple reference genotypes g_i_, processed using DeepGS models, and averaged to produce the final prediction.

We investigated the ensemble method as an approach for phenotype prediction, which we refer to as our obscured ensemble model. In this model, a single input genotype is paired with multiple other genotypes and their corresponding phenotypes. The original obscured model (Fig 4) is then applied to generate predictions, and the final prediction for the input genotype is obtained by averaging all individual predictions (see Fig 6).

The obscured ensemble model functions with the same input and output format as a typical ML model – the input to the model is the genotype information for a single individual and the output is the predicted phenotype of that individual (i.e., yield). The values of ${(g}_{i},y_{i}$) internal to the model do not vary with the input: they are fixed before training. When making a new prediction (after training), the only input is the genotype we are interested in, and the internal values remain fixed.

To train this obscured ensemble model, we use the training set values to provide the individual genotype and phenotype values within the model (i.e., the ($g_{i},y_{i}$) pairs in Fig 6). We call the instances that are used in this way to calculate the average the ***reference instances****.*

We consider whether a subset of the reference instances could be used to calculate a prediction for the trait when averaging individual predictions. By employing a subset of the training instances, we improve the efficiency of the model in producing a prediction, by only obtaining a fixed number of obscured predictions (i.e., not dependent on the input).

We consider two different techniques to reduce the set of reference instances:

(1) a random sampling method, where the reference instances are chosen at random,
(2) a targeted sampling method. In this method, each training instance’s mean squared error (MSE) for phenotype prediction is computed across all training instances. Instances with the lowest average MSE are chosen as reference instances, under the assumption that they provide more accurate predictions across the dataset.

For both approaches, different fractions of the reference instances are used.

The obscured model is initially trained once in an end-to-end manner using the entire training set. After training, the same learned parameters are used each time the obscured model is applied using any of the training data in the ensemble model.

### Evaluation metrics

To assess the performance of the obscured ensemble model, we used two standard evaluation metrics:

1. Mean Squared Error (MSE): Measures the average squared difference between predicted and actual phenotype values. Lower MSE values indicate better predictive accuracy.
2. Pearson’s Correlation Coefficient (PCC): Evaluates the linear correlation between predicted and actual phenotypes. A PCC close to 1 suggests strong agreement, while a PCC near 0 indicates no correlation.

By using both MSE and PCC, we ensure a reliable evaluation of the model’s predictive performance and its ability to capture meaningful patterns in phenotype prediction.

## Results

### Feature selection

**Fig 7:**
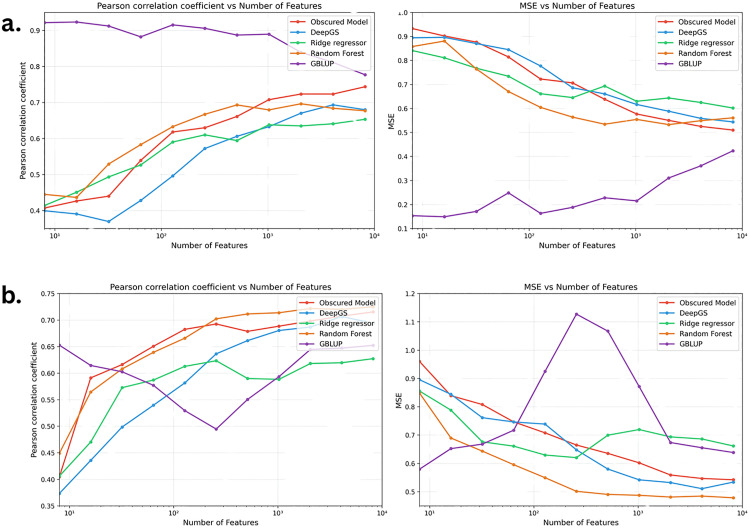
Pearson’s r and MSE versus number of features for (a) Canola Oil Content and (b) Canola Yield.

After removing redundant features, a standard filter-based feature selection method was used to further reduce the number of features down to a minimum of 8 features. Pearson correlation coefficient increased with increased number of features, while MSE decreased, for all ML models. For GBLUP, the performance was not consistent with the behaviour for the ML models, showing differing behaviours for canola yield and canola oil content. Fig 7 shows the effect overall on the PCC and MSE when increasing the number of features when predicting oil content and yield in canola. A similar trend is observed for the ML models on the lentil DTF data (Supplementary S1 and S2 Figs).

The same trend of increasing performance with increasing numbers of features is observed for all ML models tested. This supports the observations of Ubbens *et al.* that shortcut learning is occurring. As more features are added, all models continue to improve, suggesting they are finding patterns beyond just repeated or dense features. If redundancy alone caused the improvement, performance would eventually level off, but instead, it keeps increasing. The models are relying on hidden shortcuts in the data rather than truly understanding the underlying patterns. Similar findings by Ubbens et al. show that models often learn unintended correlations instead of meaningful relationships.

The performance of GBLUP in relation to the ML models shows different behaviour than the compared ML models. This reinforces the continued importance of GBLUP as a predictive model in GP. The results also demonstrate differences in predictive ability between traits on the same data set, with GBLUP showing superiority over other models independently of the number of features, while with the more complex yield trait, the predictive accuracy of GBLUP varies with the number of features, showing competitive performance at higher numbers of features.

As noted in the discussion regarding Fig 3, the density of makers may indicate a possible source of shortcut learning: higher density markers may yield information about important variation in the genome. In Fig 7, we demonstrate that this is not likely the case – the different ML models perform similarly under different marker densities. In fact, Fig 7 should not be taken as a comparison of the absolute performance of these ML models, or between the ML and GBLUP models. Instead, we use it to illustrate the similarity of the behaviours under different marker densities.

We observe that as the number of features increases beyond 1000, the correlation remains relatively stable, showing minimal variation. Similarly, MSE decreases with more features and stabilizes beyond this point. However, when the number of features drops below 1000, MSE increases more noticeably for some models, indicating a stronger dependence on feature count in this range.

**Fig 8:**
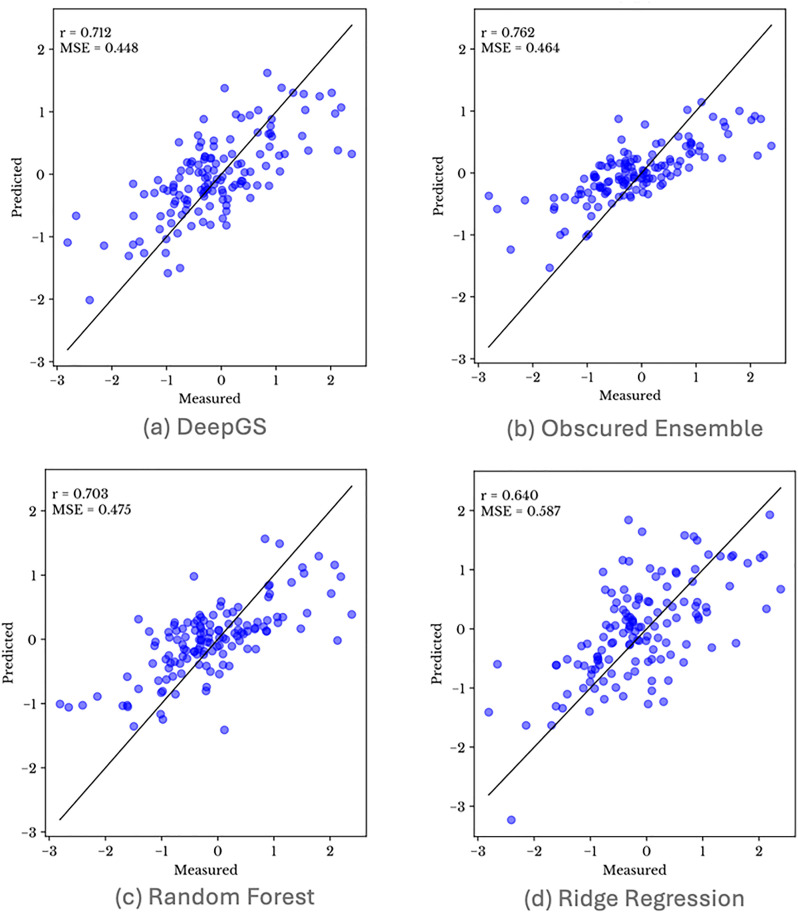
Scatter plots for a same fold of the CV for the four models (a) DeepGS, (b) Obscured Ensemble, (c) Random Forest, (d) Ridge Regression.

The cause of this discrepancy can be seen in Fig 8, which shows the scatter plots for a same fold of the cross validation for the four ML models. We can see that a linear correspondence between the actual and predicted phenotype is maintained.

### Obscured Ensemble Model Performance

**Fig 9:**
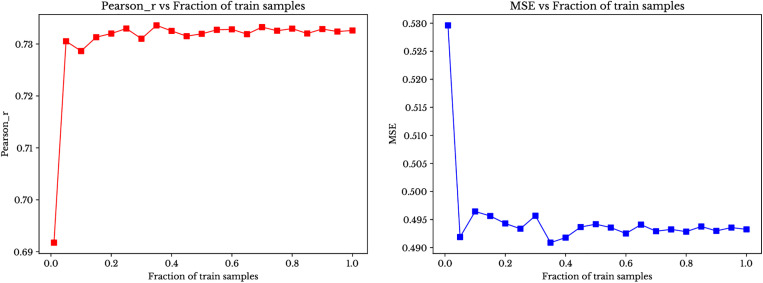
Performance with Random selection of reference instances for canola yield.

**Fig 10:**
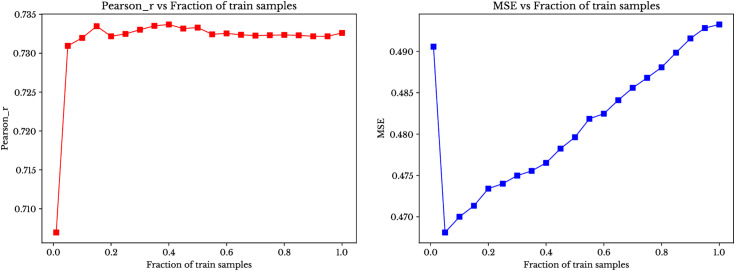
Performance with selection through target prediction accuracy for canola yield.

Model performance at all levels is shown in Fig 9 and 10. Fig 9 shows the random reference instance selection and Fig 10 shows the optimised sampling approach.

As the figures demonstrate, our ensemble model performance supports the claim that model works effectively with only 5% to 20% of the instances as reference instances. This trend indicates that adding more reference instances improves the linear relationship between predicted and actual values and enhances prediction accuracy, but this improvement is not significant beyond a minimal amount of reference instances (i.e., at least 5% to 20%).

Furthermore, an important observation from the figures is that the random selection of reference instances performs comparably to selection based on target prediction in genomic prediction (While the MSE plots in both figures may appear different in shape, this is primarily due to differences in the y-axis scale. The actual range of MSE values remains similar across both plots, and any apparent discrepancies are a result of axis scaling rather than significant differences in model performance). This suggests that, in the context of genomic prediction, randomly selecting a subset of reference instances can yield results that are just as reliable and consistent as methods that select instances based on specific target predictions. Heatmap charts demonstrate that random selection of instances is comparable with the optimized sampling techniques. In the heatmap charts (Supplementary S2, S3 and S4 Figs), the MSE of all elements of the training set (i.e., the pool of possible reference instances) are compared for all elements of the test set. We observe that similar MSE patterns are observed across most training instances. This gives one explanation for the comparable performance of the random and optimized strategies: given the similar MSE patterns for most training instances, random selection of the reference instances suffices.

## Discussion

### Importance of analyzing multiple measures

In our study, we emphasize the importance of analyzing both PCC and MSE to evaluate the efficacy of GP models. While PCC provides insight into the linear relationship between predicted and measured values, it does not fully capture the predictive accuracy and reliability of the model. Therefore, it is essential to consider MSE alongside PCC for a full assessment.

**Fig 11:**
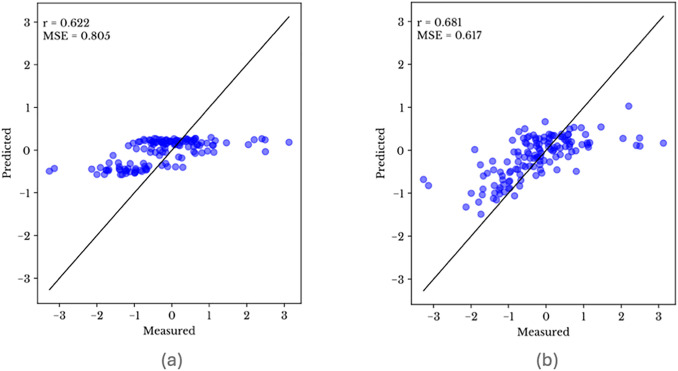
Pearson correlation (r) and Mean Squared Error (MSE) for canola dataset with (a) 32 markers and (b) 4096 markers.

As demonstrated in our analysis, we compared models using different subsets of features for the canola dataset, specifically evaluating the performance with 32 markers and 4096 markers. The results, as illustrated in Fig 11 for obscured ensemble model, clearly highlight the necessity of incorporating MSE into the evaluation process.

The relative difference in Pearson correlation coefficient and MSE between the two sets is important to note. For the model using 32 markers, although the Pearson correlation values indicate a moderate to strong correlation (0.622), the scatter plot reflects poor prediction, with relatively constant predicted values. In contrast, the model using 4096 markers shows a Pearson correlation coefficient value (0.681) that is similar to the previous experiment with 32 markers. However, the scatter plot reveals an improvement in the range of predicted yield values. However, the MSE change between the two experiments (from 0.805 to 0.617) illustrates that the more accurate and reliable prediction reflects the improvement visible in the scatterplot.

These findings underscore the limitation of relying solely on Pearson correlation coefficient when evaluating predictive models. A high Pearson correlation coefficient does not necessarily imply good predictive performance if the MSE is high, as it indicates larger prediction errors. Therefore, for a thorough and accurate evaluation of phenotype prediction models, it is imperative to consider both Pearson correlation coefficient and Mean Squared Error.

### Selecting reference instances

The number of reference instances used in the obscured ensemble method can be considered analogous to other hyperparameters in supervised ensemble learning models. For instance, the number of trees in a random forest is one of hyperparameter that users may consider modifying, with the understanding that more trees generally increase performance. However, Oshio et al. [31] and Probst and Boulesteiux [32] demonstrate on several data sets that there is a plateau on the number of trees, and exceeding these values may lead to reduced prediction success. They state that using 100 trees typically leads to successful results, regardless of the number of instances in the training set.

Similarly, our results show that the number of reference instances in the obscured ensemble method may also be a hyperparameter where increasing the fraction of the training set used does not necessarily improve results once it grows towards 1. Further, choosing those reference instances that yield the best predictions does not tend to provide significant improvement over randomly selecting reference instances.

For the obscured ensemble model, we hypothesized that a targeted set of reference instances would provide the most accurate basis for the ensemble model: the best performing reference instances in the training set could be expected to generalize to prediction on unseen instances in the test set. However, our results demonstrate similar performance can be obtained through a random selection of reference instances. This suggests that genomic variation, sufficient to create a diversity of obscured vectors for the ensemble model, can be obtained with relatively basic sampling. This leads to possible insights into the nature of the diversity needed to cover the genomic spaces sufficiently for an ensemble-type prediction. Further investigation could investigate further selection techniques and their relationship with, for example, population structure.

There are analogies between our method, which represents similarities between genomes through the obscured vector, and GBLUP, which represents similarities between genomes through the genomic relationship matrix (GRM). The GRM represents these similarities as a single number, while the obscured vector represents this similarity in a richer way. Similar techniques for GBLUP to produce a smaller GRM may be considered.

Beyond the numerical improvements, the reduction in MSE and sustained high correlation achieved by our proposed approach have meaningful implications for genomic selection in crops breeding. Lower prediction errors translate directly into more reliable selection decisions, enabling breeders to focus resources on top-performing genotypes with greater confidence. In turn, this can expedite the breeding cycle and reduce costs associated with extensive field evaluations. For instance, even modest improvements in prediction accuracy can yield substantial gains over multiple selection cycles, ultimately improving yield, disease resistance, and other agronomic traits. Nevertheless, it is important to note that these gains should be validated across diverse environments and breeding programs to confirm their robustness. Furthermore, integrating environmental covariates or multi-trait prediction could further enhance the real-world utility of our method.

## Conclusion

In this paper, we have demonstrated that the performance of the four ML models, including the obscured model, is similar under different numbers of selected features. The performance decreases, as expected, as the number of features decreases. This suggests that the shortcut learning observed by Ubbens *et al.* can be attributed to general GP, rather than to linkage specifically. If linkage were the cause of the shortcut learning, we would expect that performing feature selection (including the removal of redundant features) would disproportionately affect the performance of the obscured model. These observations can drive insight and best practices around the sparsity of genomic markers and feature selection in genomic selection, especially of polygenic traits.

Our results demonstrate prediction of phenotype using approximately 20% of the markers from each reference genotype. By strategically identifying and employing the most informative subset of features, our method reduces computational complexity of training and improves prediction speed. The approximate linearity of Pearson correlation coefficient and MSE beyond the 20% marker threshold indicates that increasing the number of markers does not significantly enhance predictive accuracy.

Our results on obscured ensemble methods demonstrate that these models have potential as a method for GP. A single DL model is trained and similarity to a subset of reference instances is used to enable prediction using this DL model. Average prediction of all predictions using the obscured model shows promise as a GP model.

## Supporting information

S1 Fig Pearson’s r and MSE versus number of features for Lentil DTF. (TIF)

S2 Fig Heatmap of MSE of training versus test instances for canola yield. (TIF)

S3 Fig Heatmap of MSE of training versus test instances for canola oil content. (TIF)

S4 Fig Heatmap of MSE of training versus test instances for lentil DTF. (TIF)

S1 File Genotype and phenotype data for canola dataset. (CSV)
