# Supplementary figures and images for: Obscured-ensemble models for genomic prediction
Source: PLoS One. 2025 Nov 14;20(11):e0334239. doi: 10.1371/journal.pone.0334239 (PMC12617858; doi:10.1371/journal.pone.0334239)

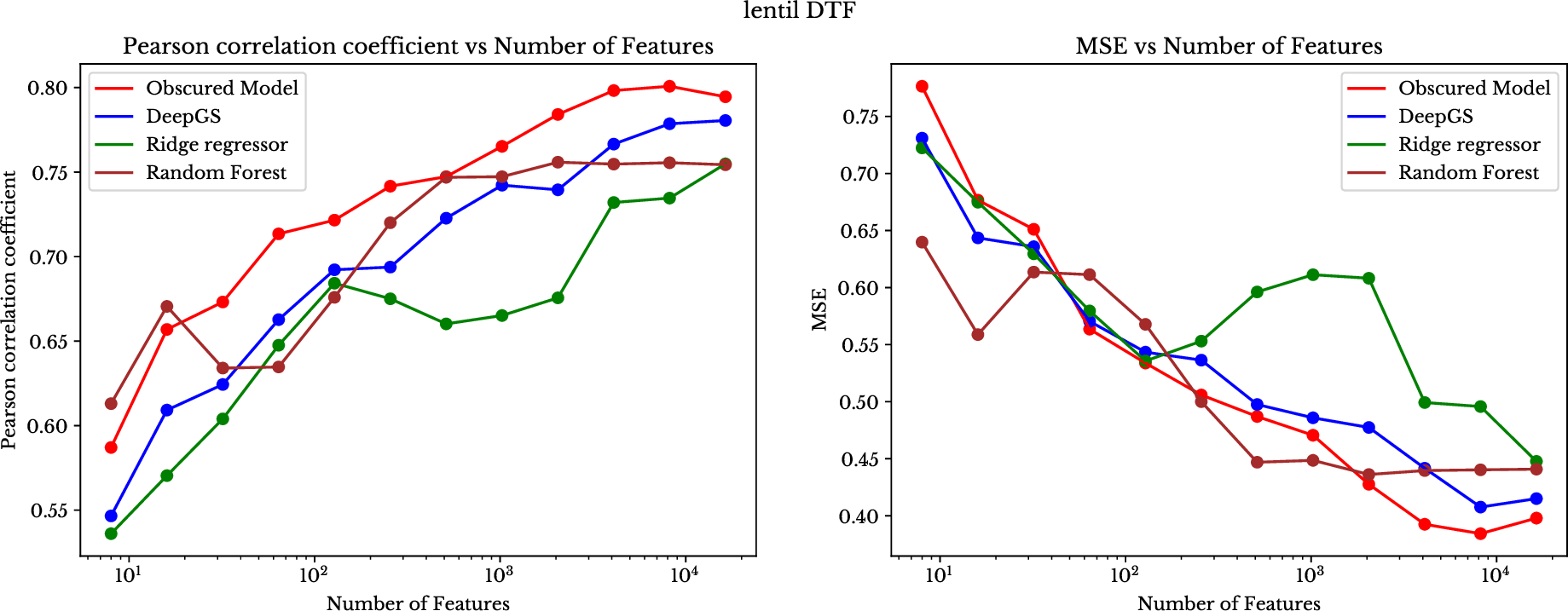

Supplement: S1 Fig — (TIF) [file pone.0334239.s001.tif]

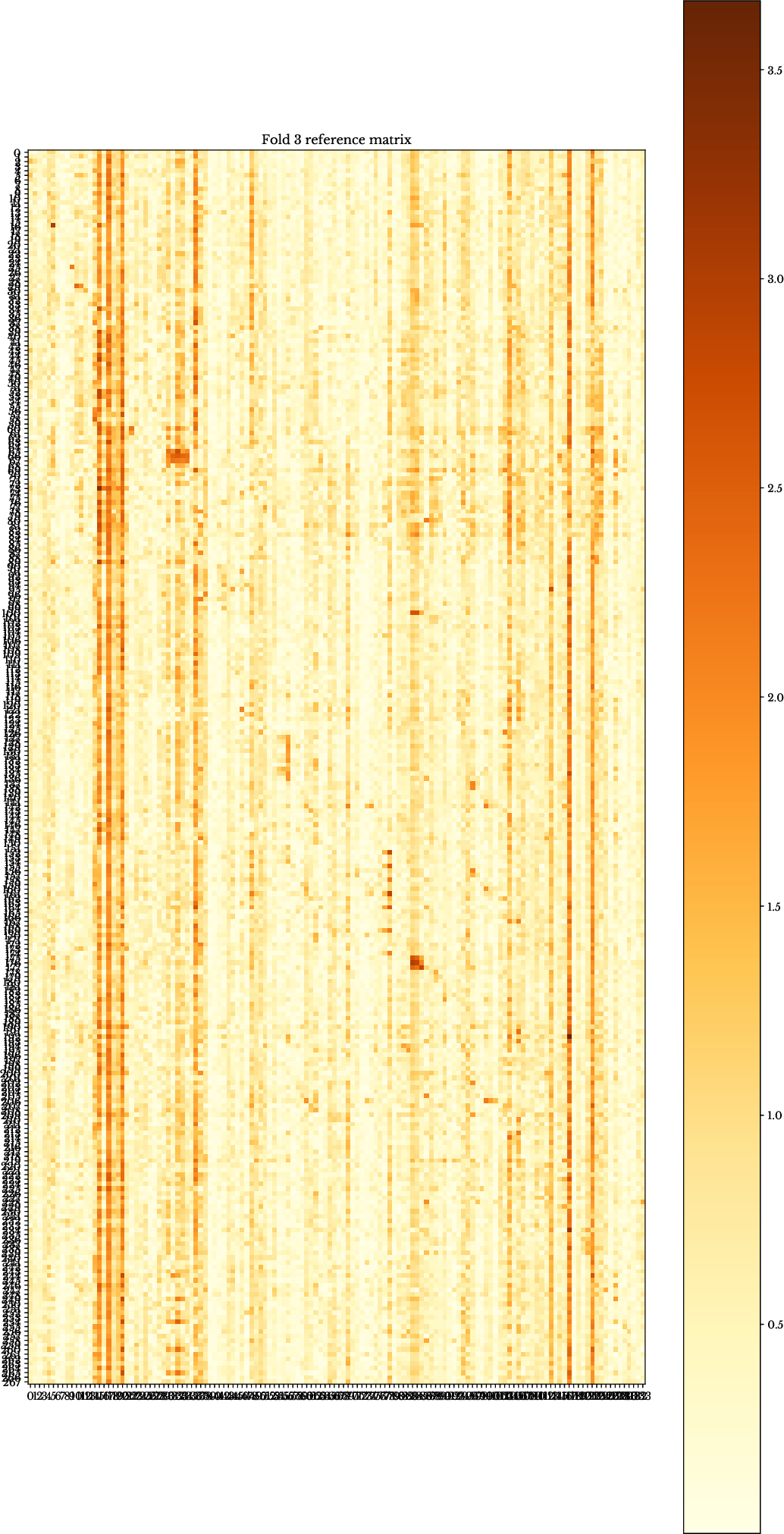

Supplement: S2 Fig — (TIF) [file pone.0334239.s002.tif]

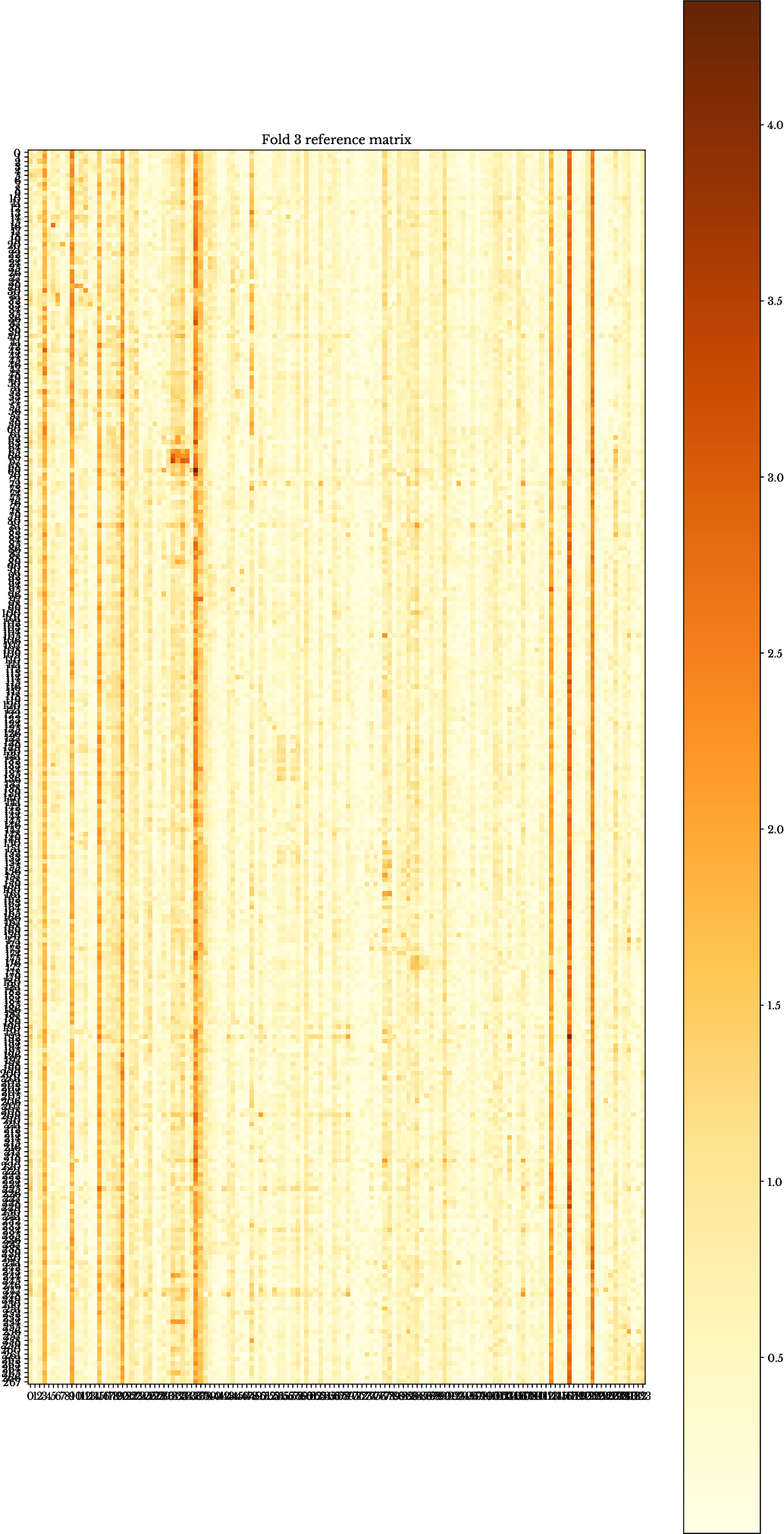

Supplement: S3 Fig — (TIF) [file pone.0334239.s003.tif]

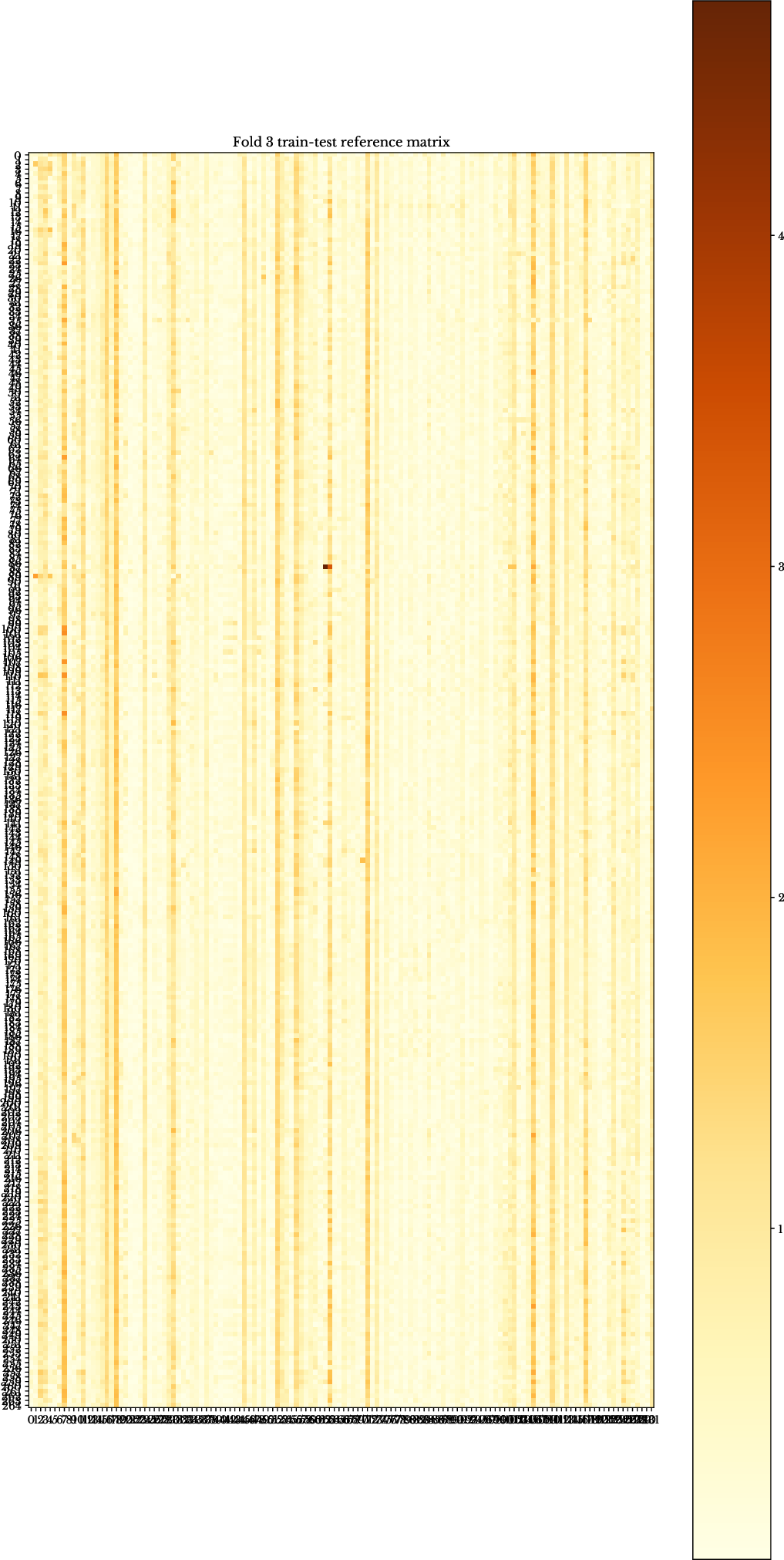

Supplement: S4 Fig — (TIF) [file pone.0334239.s004.tif]
